# Structural Basis for the Interaction of the Adaptor Protein Grb14 with Activated Ras

**DOI:** 10.1371/journal.pone.0072473

**Published:** 2013-08-13

**Authors:** Rohini Qamra, Stevan R. Hubbard

**Affiliations:** Kimmel Center for Biology and Medicine of the Skirball Institute and Department of Biochemistry and Molecular Pharmacology, New York University School of Medicine, New York, New York, United States of America; University of Washington, United States of America

## Abstract

Grb14, a member of the Grb7-10-14 family of cytoplasmic adaptor proteins, is a tissue-specific negative regulator of insulin signaling. Grb7-10-14 contain several signaling modules, including a Ras-associating (RA) domain, a pleckstrin-homology (PH) domain, a family-specific BPS (between PH and SH2) region, and a C-terminal Src-homology-2 (SH2) domain. We showed previously that the RA and PH domains, along with the BPS region and SH2 domain, are necessary for downregulation of insulin signaling. Here, we report the crystal structure at 2.4-Å resolution of the Grb14 RA and PH domains in complex with GTP-loaded H-Ras (G12V). The structure reveals that the Grb14 RA and PH domains form an integrated structural unit capable of binding simultaneously to small GTPases and phosphoinositide lipids. The overall mode of binding of the Grb14 RA domain to activated H-Ras is similar to that of the RA domains of RalGDS and Raf1 but with important distinctions. The integrated RA-PH structural unit in Grb7-10-14 is also found in a second adaptor family that includes Rap1-interacting adaptor molecule (RIAM) and lamellipodin, proteins involved in actin-cytoskeleton rearrangement. The structure of Grb14 RA-PH in complex with H-Ras represents the first detailed molecular characterization of tandem RA-PH domains bound to a small GTPase and provides insights into the molecular basis for specificity.

## Introduction

Grb14 is a member of the Grb7-10-14 family of multi-domain, cytoplasmic adaptor proteins. These proteins are recruited to activated receptor tyrosine kinases, including the insulin receptor, insulin-like growth factor-1 (IGF1) receptor, fibroblast growth factor receptor-1, and members of the epidermal growth factor receptor family [1]. Gene-deletion and transgenic studies in mice established that Grb10 and Grb14 are tissue-specific, negative regulators of insulin action [2–6]. Male Grb14^−/−^ mice exhibit improved glucose tolerance and enhanced insulin signaling in muscle and liver [2]. In the ob*/*ob mouse model for non-insulin-dependent (type II) diabetes, Grb14 mRNA levels are increased by 75%-100% in adipose tissue [7]. In human type II diabetic patients, Grb14 mRNA levels were elevated by 43% in subcutaneous adipose tissue compared with non-diabetic control patients [7]. *Grb10* is imprinted in mice [8] (and most likely in humans [9]), and loss of the maternal allele results in mice that are approximately 30% greater in overall size than wild-type litter mates, with disproportionately large livers [6]. As adults, these mice exhibit improved glucose tolerance, increased muscle mass, and reduced adiposity [3]. Transgenic mice overexpressing Grb10 show postnatal growth retardation and insulin resistance as a consequence of hyper-negative regulation of the insulin and IGF1 receptors [5].

Grb7-10-14 share a common domain architecture, possessing an N-terminal polyproline region, a Ras-associating (RA) domain (also known as a Ras binding domain (RBD)), a pleckstrin-homology (PH) domain, a family-specific BPS (between PH and SH2) region, and a C-terminal Src-homology-2 (SH2) domain. Previous structural and biochemical studies of Grb14 established that: (i) the BPS region inhibits the catalytic activity of the insulin receptor [10] by binding as a pseudosubstrate in the kinase active site [11]; (ii) the SH2 domain binds directly to the phosphorylated activation loop of the insulin receptor kinase domain [11]; and (iii) functional RA and PH domains are required for negative regulation of the insulin receptor [12].

We also showed that Grb14 and Grb7 (Grb10, much less so) could interact with activated N-Ras in transfected cells and hypothesized that Ras activation could serve as a timing mechanism for negative-feedback regulation of the insulin receptor by Grb14 [12]. The small GTPases N-Ras, H-Ras, and K-Ras serve as molecular switches in cellular growth control, cycling between a GDP-bound inactive state and a GTP-bound active state, and gain-of-function mutations in the Ras proteins are found in approximately 30% of human cancers [13].

A second family of adaptor proteins, which includes Rap1-interacting adaptor molecule (RIAM) [14] and lamellipodin [15], is involved in actin-cytoskeleton rearrangement. These adapter proteins also contain tandem RA and PH domains with high sequence similarity to those in Grb7-10-14 [16]. RIAM has been shown to be recruited to activated Rap1, a small GTPase related to Ras, at the plasma membrane [14,17], although the specificity determinants for the interaction of RA-PH proteins with small GTPases are largely unknown.

To understand the molecular basis for the interaction of Grb14 with activated Ras, we determined the crystal structure of the Grb14 RA and PH domains in complex with activated H-Ras and measured the *in vitro* binding affinity of this interaction. These data provide a molecular basis for understanding the specificity determinants that govern the binding of Grb14 to Ras and, more generally, of RA-PH proteins to small GTPases at the plasma membrane.

## Results

### Co-crystal structure of Grb14 RA-PH and H-Ras

**Figure 1:**
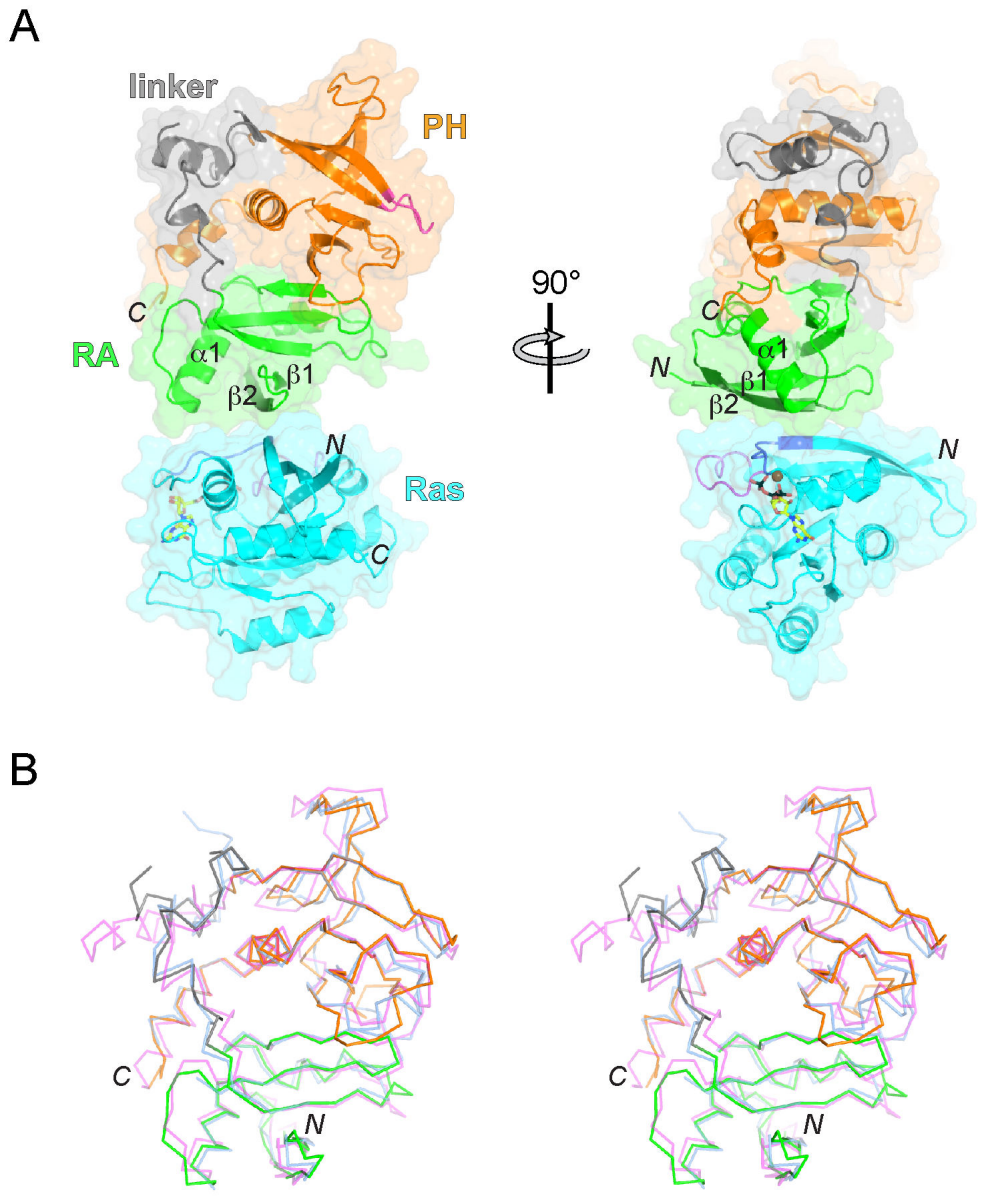
Crystal structure of Grb14^RA-PH^-H-Ras and comparison to other RA-PH structures. (A) Ribbon diagram and molecular surface representation of the Grb14^RA-PH^-H-Ras complex. The Grb14 RA domain is colored green, with β2 colored dark green, the PH domain is colored orange, with the phosphoinositide-binding β1-β2 loop colored magenta, and the RA-PH linker is colored gray. H-Ras is colored cyan, with switch 1 (residues 32-38) colored purple and switch 2 (residues 59-67) colored magenta. GTP is shown in stick representation with carbon atoms colored yellow, oxygen atoms red, nitrogen atoms blue, and phosphorus atoms black. The Mg^2+^ ion is colored brown. Select secondary-structure elements are labeled, along with the N- and C-termini. The view on the right is rotated by 90° as indicated. (B) Stereo diagram of a superposition of the structures (Cα traces) of Grb14^RA-PH^, Grb10^RA-PH^ (PDB code 3HK0) [12], and RIAM^RA-PH^ (PDB code 3TCA) [17], in the same orientation as in the left panel of (A). Grb14^RA-PH^ is colored by domain as in (A), Grb10^RA-PH^ is colored blue (all) and RIAM^RA-PH^ is colored magenta (all). The N- and C-termini are labeled.

We determined the crystal structure of a complex between Grb14 RA-PH (Grb14^RA-PH^) and a constitutively active mutant (G12V) of H-Ras loaded with Mg-GTP. We determined the structure by molecular replacement, using the structures of Grb10^RA-PH^ [12] and nucleotide-loaded H-Ras [18] as search models. Data collection and refinement statistics at 2.4-Å resolution appear in Table 1. The overall structure of the complex is shown in Figure 1A. As first observed in the Grb10^RA-PH^ structure [12], and more recently in the RIAM [17] and lamellipodin [19] RA-PH structures, the RA and PH domains of Grb14, together with the intervening linker of ~40 residues, share an extensive interaction interface, which creates an integrated two-domain structural unit. The four copies of Grb14^RA-PH^-Ras in the asymmetric unit are highly similar and superimpose on one another with a root-mean-square deviation (rmsd) of <1 Å (410 Cα atoms).

**Table 1:** X-ray data collection and refinement statistics.

*Data collection*	
X-ray wavelength	1.000
Space group	*P21*
Unit cell parameters	
a, b, c (Å)	79.7, 115.6, 103.1
α, β, γ (°)	90.0, 96.75, 90.00
Resolution (Å)	50.0-2.4
No. of observations	266,792
No. of unique reflections	71,989
Redundancy	3.7 (3.2)*
Completeness (%)	99.9 (99.6)*
*R*_sym_	5.8 (36.6)*
*<I*/σ*I>*	23.8 (2.2)*
*Refinement*	
Resolution (Å)	50.0-2.4
Number of atoms	
Protein	13,500
Ligand	186
Solvent	181
No. of reflections total	68,338
No. of reflections test set	3,628 (5%)
*R*_cryst_ / *R*_free_	21.8/27.7
r.m.s.d values	
Bond lengths (Å)	0.008
Bond angles (°)	1.23
Average *B*-factors (Å^2^)	
All atoms	66.5
Protein	66.9
Ligand	53.9
Solvent	43.2
Ramachandran plot statistics (%)	
Most favored	88.7
Additionally allowed	10.8
Generously allowed	0.5
Disallowed	0

* Values in parentheses are for the highest-resolution shell. One crystal was used for the data set.

One TLS (translation/liberation/screw) parameter for each molecule was included in the refinement. Atomic coordinates and structure factors have been deposited in the Protein Data Bank with accession number 4K81.

A comparison of the Grb14^RA-PH^ structure with those of Grb10^RA-PH^ and RIAM^RA-PH^ shows that the integrated RA-PH structural unit is well preserved, with the main differences found in the RA-PH linker. Superposition of Grb14^RA-PH^ with Grb10^RA-PH^ [12] (Figure 1B) yields an overall rmsd of 3.0 Å (227 Cα atoms superimposed) or 2.1 Å (192 Cα atoms) if the RA-PH linker is excluded. For the individual domains, the rmsd values are 1.3 Å for the RA domain (75 Cα atoms) and 2.5 Å for the PH domain (116 Cα atoms). Comparing Grb14^RA-PH^ to RIAM^RA-PH^ [17] (Figure 1B), the rmsd values are 2.7 Å overall (201 Cα atoms; the RA-PH linker excluded), 1.1 Å for the RA domain (82 Cα atoms), and 3.1 Å for the PH domain (116 Cα atoms).

### Comparison to other RA domain-Ras structures

Despite low sequence identity (<20%), the Grb14 RA domain adopts the same ubiquitin-like fold of the RA domains (RBDs) of RalGDS [18] and Raf1 [20], consisting of a five-stranded β sheet and two α helices. The PH domain adopts the canonical PH-domain fold [21], comprising a seven-stranded β sheet and a C-terminal α helix. An additional helix follows on the C-terminal end, which is also present in the Grb10 [12], RIAM [17], and lamellipodin [22] RA-PH structures. In the Grb10 RA-PH structure, this extra helix mediates RA-PH dimerization [12], but it is not observed to do so in the Grb14 RA-PH structure, although that does not necessarily preclude a dimerization role for this helix *in vivo*.

**Figure 2:**
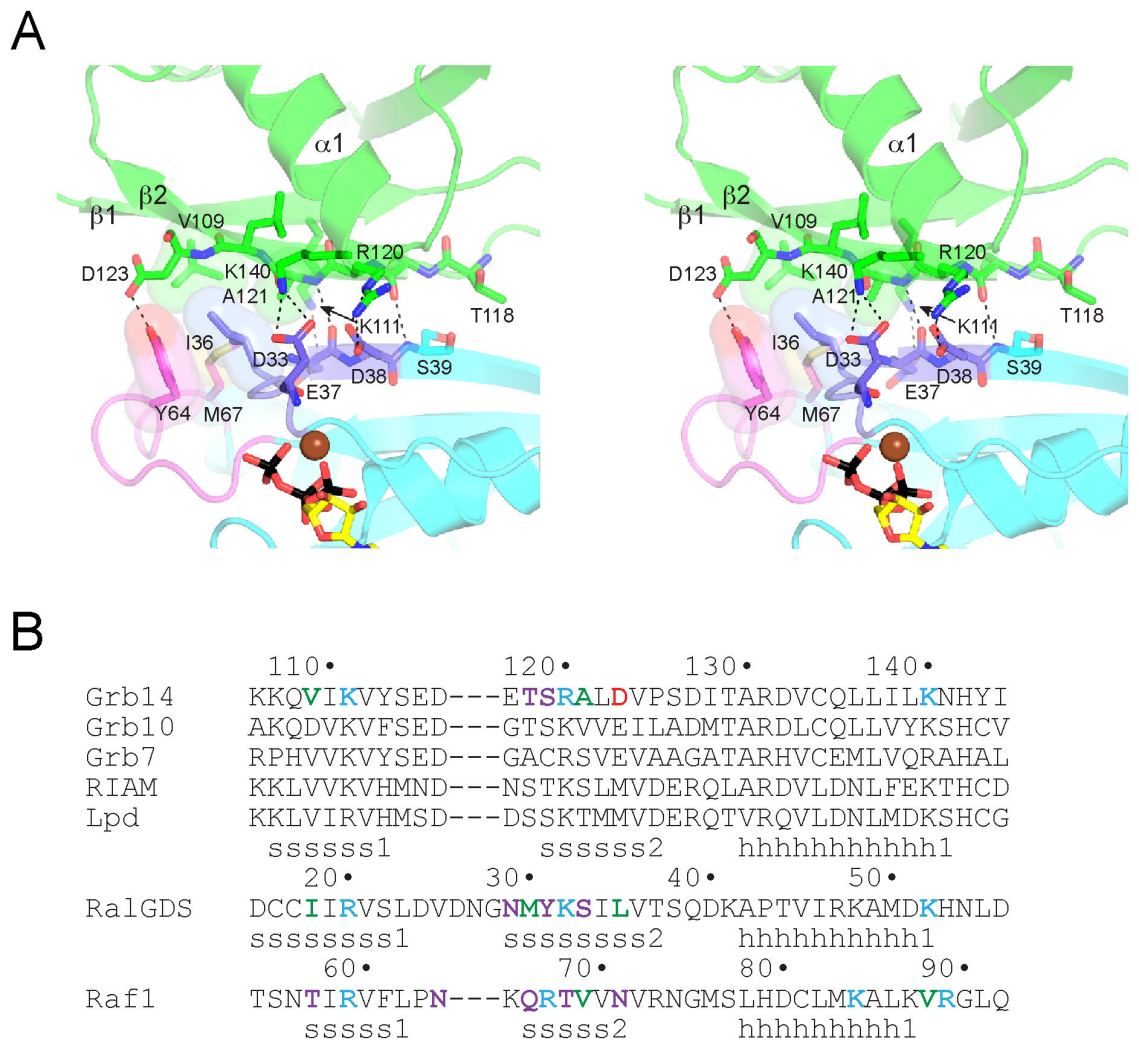
Mode of binding of Grb14^RA-PH^ to H-Ras. (A) Stereo diagram of the binding interface. The Grb14 RA domain is colored green, and H-Ras is colored cyan, with switch 1 colored purple and switch 2 colored magenta. Select side-chain and backbone atoms are shown in stick representation, with oxygen atoms colored red and nitrogen atoms colored blue. Mg-GTP is colored as in Figure 1. Select hydrogen bonds and salt bridges are represented by black dashed lines, and van der Waals interactions by side-chain surfaces. (B) Structure-based sequence alignment of RA domains. The top set of sequences (human) are for the RA-PH-containing proteins: Grb7-10-14, RIAM, and lamellipodin (Lpd). Residues that interact with Ras (or Rap1 in the case of Raf1) are colored red for acidic, cyan for basic, violet for non-charged polar, or green for hydrophobic. The secondary structure assignments (s, β strand; h, α helix) for the three structures are shown below the sequences.

Grb14^RA-PH^ interacts with GTP-loaded H-Ras (G12V) exclusively via the RA domain (Figures 1A, 2A), with the β1-β2 loop of the PH domain free to bind to the headgroup of membrane phosphoinositides [21] (Figure 1A). The mode of engagement with Ras is similar to that of other RA domains interacting with Ras, including those of RalGDS [18], phosphoinositide 3-kinase [23], phospholipase Cε [24], and Nore1 [25], or the RA domain of Raf1 [20,26] interacting with Rap1. A common feature of RA-domain binding to activated Ras is backbone hydrogen-bonding between β2 of the RA domain and β2 in the switch 1 region of Ras. This interaction is fortified by side-chain interactions involving residues in β1, β2, and α1 of the RA domain and residues in the switch 1 (predominantly) and 2 regions of Ras. Specifically, Lys111 (β1) of Grb14^RA-PH^ is salt-bridged to Glu37 (switch 1) of Ras, Arg120 (β2) is salt-bridged to Asp38 (switch 1), Asp123 (β2) is hydrogen-bonded to Tyr64 (switch 2), and Lys140 (α1) is salt-bridged to Asp33 (switch 1) (Figure 2A). A small hydrophobic cluster is formed between residues Val109 (β1) and Ala121 (β2) of Grb14^RA-PH^ and Ile36 (switch 1), Tyr64 (switch 2), and Met67 (switch 2) of Ras. The Grb14^RA-PH^-Ras complex buries a modest total surface area of 890 Å^2^.

The Grb14^RA-PH^-Ras structure provides a rationale as to why Ras binds more tightly to Grb14 and Grb7 than to Grb10 [12]. In place of Val109 (β1) in Grb14 is an aspartic acid in Grb10 (Figure 2B), which would not contribute to the hydrophobic cluster described above. Valine is conserved at this position in Grb7, RIAM, and lamellipodin. Two other differences that probably adversely affect Ras binding to Grb10 are Arg120 (β2) (lysine in Grb10) and Asp123 (β2) (glutamic acid in Grb10), because arginine typically forms stronger salt bridges than lysine [27], and glutamic acid, with its longer side chain, would appear to be less capable than Asp123 of hydrogen bonding with Tyr64 of Ras. It is not clear whether these amino-acid differences in Grb10 confer specificity for some other small GTPase or whether, in general, Grb10 has a diminished capacity for binding to GTPases.

**Figure 3:**
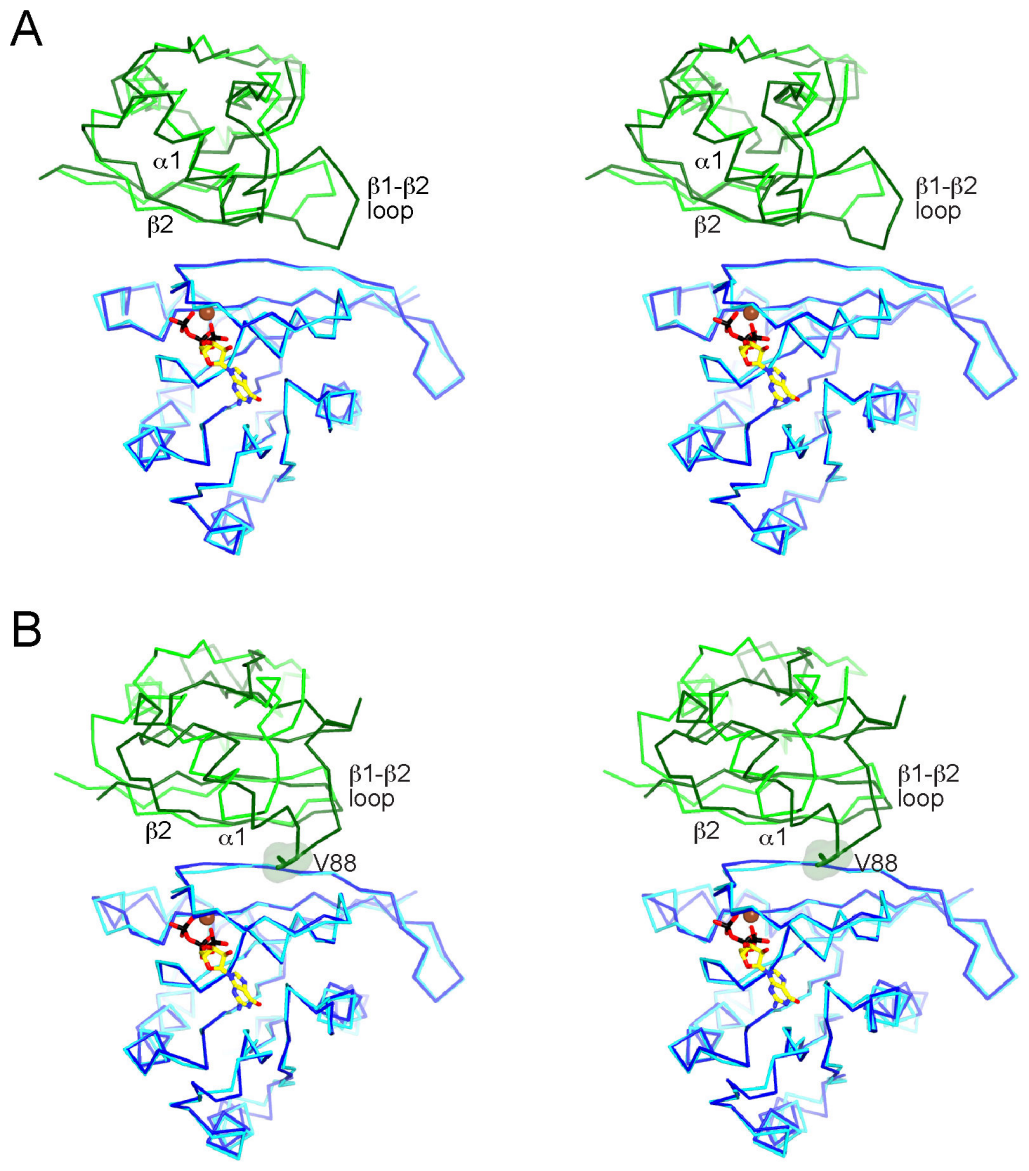
Comparison of Grb14^RA-PH^-H-Ras structure with other RA domain-GTPase structures. (A) Stereo diagram of a superposition (aligned on Ras) of the structures (Cα traces) of Grb14^RA-PH^-H-Ras and RalGDS^RA^-H-Ras (PDB code 1LFD) [18]. Grb14^RA^ is colored green, RalGDS^RA^ is colored dark green, H-Ras is colored cyan (for Grb14^RA^) or dark blue (for RalGDS^RA^). Mg-GTP is shown bound to H-Ras in the Grb14^RA-PH^ structure. Select secondary-structure elements in the RA domains are labeled. (B) Stereo diagram of a superposition (aligned on Ras/Rap1) of the structures of Grb14^RA-PH^-H-Ras and Raf1^RA^-Rap1 (PDB code 1GUA) [26]. Raf1^RA^ is colored dark green and Rap1 is colored dark blue. Val88 of Raf1^RA^ is shown in stick and surface representation.

A superposition of the structures of Grb14^RA-PH^-Ras and RalGDS^RA^-Ras [18] shows a similar mode of Ras engagement (Figure 3A). The conformation of nucleotide-loaded Ras is virtually identical, with an rmsd of 0.5 Å (166 Cα atoms). Mg-GTP is bound canonically to Ras in the Grb14^RA-PH^-Ras structure. The RA domains superimpose reasonably well, in particular, β1, β2, and α1. There is a three-residue insertion in the β1-β2 turn in RalGDS^RA^ relative to Grb14^RA-PH^, which results in additional backbone (Gly28) and side-chain (Asn29) interactions between β2 of RalGDS^RA^ and Ras. The RalGDS^RA^-Ras complex buries a total surface area of 1200 Å^2^.

Ras is the physiologic target of the Raf1 RA domain, yet a co-crystal structure of Raf1^RA^ with GTP-bound Ras has not been reported, although a structure with GDP-bound Ras has been [28]. In the earlier crystallographic studies of Raf1^RA^, Rap1 was used as a surrogate for Ras, without [20] or with [26] amino-acid substitutions that make Rap1 more Ras-like. The structure of Raf1^RA^-Rap1 superimposed on the structure of Grb14^RA-PH^-Ras shows that the interactions between β1 and β2 of the RA domains with switch 1 of the GTPases are similar, but that the position of α1 in the RA domains is divergent (Figure 3B). In the Raf1^RA^-Rap1 structure (total buried surface area of 1310 Å^2^), residues at the end of α1 make contact with Rap1, in particular, Val88 with Val21 of Rap1 (Ile21 in Ras) and Arg89 with Asp38 (same in Ras). Based on the lack of conservation of a hydrophobic residue corresponding to Val88 of Raf1^RA^ (Figure 2B), these additional α1 interactions are predicted to be lacking for the RA-PH domains of Grb7-10-14, RIAM, and lamellipodin, as they are for the RalGDS RA domain [18]. Because these α1 interactions are also observed in the structure of Raf1^RA^ bound to GDP-loaded Ras [28], the difference in α1 behavior is not due to engagement of Rap1 versus Ras.

### In vitro binding affinity of Grb14 RA-PH and H-Ras

**Figure 4:**
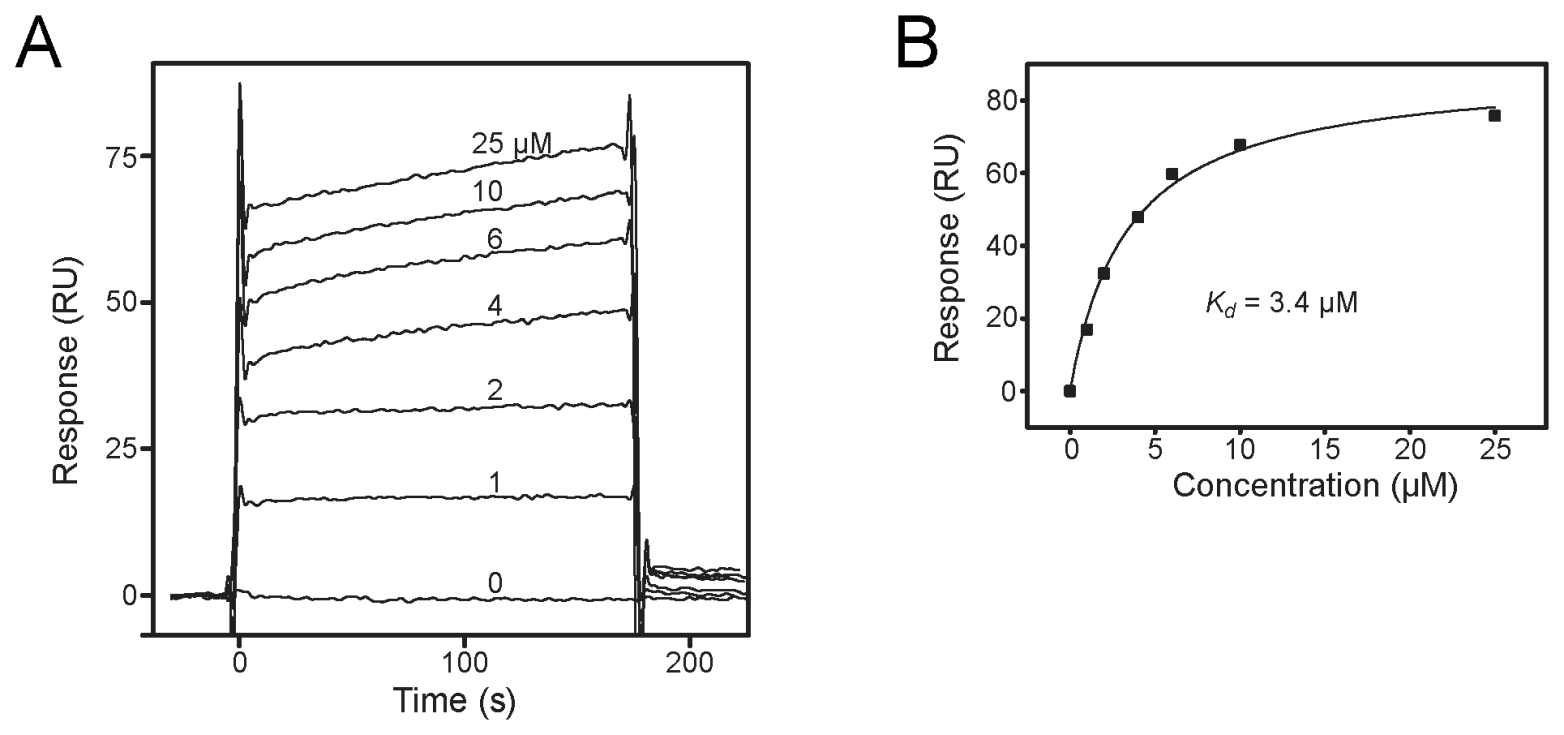
Measurement of the *in vitro* binding affinity of Grb14^RA-PH^ and H-Ras. (A) Representative (from one of three independent experiments) SPR sensorgrams (response units (RU) versus time) are shown for Grb14^RA-PH^, at the concentrations indicated, flowing over a biosensor chip containing immobilized GST-H-Ras (G12V) loaded with Mg-GTP. (B) The average response from 130–160 s was plotted as a function of Grb14^RA-PH^ concentration and fitted to a steady-state model to calculate a *K*_*d*_ of 3.4 µM. The average *K*_*d*_ calculated from three independent experiments was 3.6 ± 0.6 µM.

We performed surface plasmon resonance (SPR) experiments to determine the *in vitro* binding affinity of Grb14^RA-PH^ for Ras (Figure 4). We prepared GTP-loaded GST-H-Ras (G12V), coupled this protein to the biosensor chip, and flowed various concentrations of Grb14^RA-PH^ over the chip. Data from three independent experiments yielded an average dissociation constant (*K*_*d*_) of 3.6 ± 0.6 µM. For comparison, we also determined the dissociation constant for Raf1^RA^ binding to GST-H-Ras (G12V) (data not shown). The measured *K*_*d*_ of 50 nM is considerably lower and in the same range as measured previously (80 nM) by isothermal titration calorimetry [29]. The binding affinity between RalGDS^RA^ and activated Ras was reported to be 1.9 µM [30], which is comparable to the Grb14^RA-PH^ affinity.

## Discussion

Based on crystal structures and sequence alignments, the integrated RA-PH structural module (Figure 1A) is now firmly established in the Grb7-10-14 family of adaptor proteins and in a distinct adaptor-protein family that includes RIAM and lamellipodin, which are involved in actin-cyto-skeleton rearrangement [16]. In this integrated module, the RA and PH domains are configured to bind independently (i.e., there is no steric occlusion) to their respective targets, small GTPases and phosphoinositides (Figure 1A). Moreover, the PH domain can potentiate binding of the RA domain to GTPases tethered to the plasma membrane by assisting in membrane targeting. We showed previously that a functional PH domain was required for the co-immunoprecipitation from cells of Grb14 with activated N-Ras [12].

The overall mode of binding of Grb14^RA-PH^ to activated Ras is similar to that of Raf1^RA^ and RalGDS^RA^, mediated primarily by residues in β2 of the RA domain and in switch 1 of Ras (Figure 2A), yet an important difference in the binding modes is evident. For Grb14^RA-PH^ and RalGDS^RA^, a single residue in α1 (Lys140 in Grb14) contributes to Ras binding, whereas for Raf1^RA^, because of the distinct positioning of α1 on the Ras surface (Figure 3B), three residues in the helix (or just beyond) contact Ras. This difference evidently accounts for the higher affinity of Raf1^RA^ binding to Ras (*K*_*d*_ ~ 80 nM) [29], relative to Grb14^RA-PH^ (3.6 µM) and RalGDS^RA^ (1.9 µM) [30].

For RA-PH proteins, selectivity for activated Ras vs. Rap1 (or related GTPases) is not altogether clear. We showed previously that Grb14 does not bind appreciably to Rap1 relative to Ras [12]. In a study of the RIAM RA-PH domains [17], it was shown that Rap1 was the target of the RA domain in cells, where Ras was also present, yet *in vitro*, RIAM^RA-PH^ bound equally well to Ras. From examination of the Grb14^RA-PH^-Ras structure and sequence alignments (Figure 2A,B), two residue differences in Ras versus Rap1 could explain the preference of Grb14^RA-PH^ for Ras. Firstly, in the structure, Tyr64 in switch 2 of Ras is hydrogen-bonded to Asp123 (β2) in Grb14^RA-PH^ (Figure 2A). In Rap1, the corresponding residue is Phe64. Secondly, Glu31 of Ras (switch 1) is just outside (3.6 Å) hydrogen-bonding range of His142 in Grb14^RA-PH^ (not shown), at the end of α1. In Rap1, the equivalent residue is Lys31, which would not form a favorable interaction (at neutral pH) with His142.

Although Grb14 can interact with activated Ras, as demonstrated by co-immunoprecipitation studies [12], *in vitro* binding studies (Figure 4), and a crystal structure (Figure 1A), we do not know definitively whether Ras is the small-GTPase target of the Grb14 RA domain *in vivo*, i.e., in insulin-responsive tissues. Point mutation (K140A) of the Grb14 RA domain abrogated the inhibitory effect of Grb14 on insulin signaling [12], indicating that a functional RA domain is required. Ras is a plausible Grb14-interaction candidate because it can be activated through phosphorylation (on a Grb2 site) of insulin receptor substrate-1 (IRS1) and IRS2, the major substrates of the insulin receptor. Thus, Ras activation could serve as a trigger for downregulation of insulin signaling by facilitating recruitment of Grb14 to the activated insulin receptor.

Structurally coupling an RA domain to a PH domain could optimize positioning of the RA domain for engagement with GTPases that, like Ras and Rap1, are tethered to the plasma membrane through C-terminal prenylation [31]. In a study of the recruitment of the RIAM RA-PH domains to the plasma membrane [17], a molecule with tandem RA-PH units in which the RA domain in one unit and the PH domain in the other unit were inactivated by point mutation—effectively creating an RA domain-PH domain “beads-on-a-string” scenario (the individual domains are poorly expressed)—was less efficient in co-localizing RIAM to activated Rap1 than a single, wild-type RA-PH unit.

Moreover, in the RIAM study [17], although RalGDS^RA^ bound to Rap1 both on the plasma membrane and on endomembranes, RIAM^RA-PH^ was co-localized with Rap1 only at the plasma membrane. We showed previously that the Grb10 and Grb14 PH domains, especially the latter, bind weakly to phosphoinositides and relatively non-specifically [12], as does the RIAM PH domain [17]. Thus, by coupling a weak-binding RA domain to a non-selective and weak-binding PH domain, which favors binding to the relatively abundant PI(4,5) P_2_ in the plasma membrane, RA-PH-containing proteins can be recruited to specific small GTPases exclusively at the plasma membrane. 

## Materials and Methods

### Protein expression and purification

The cDNA encoding residues 106-356 (RA and PH domains) of human Grb14 was subcloned into a modified expression vector pET28 (Novagen) containing an N-terminal 6xHis-tag. Two mutations were introduced to facilitate crystal packing: K272A and E273A. The corresponding mutations had been introduced into Grb10^RA-PH^ for its structure determination [12]. The recombinant plasmid was transformed into *E. coli* Rosetta 2 (DE3) (Novagen), and cultures were grown in Luria Broth (LB) media supplemented with kanamycin (50 μg/ml) at 37 °C and induced with 0.2 mM isopropyl-thiogalactopyranoside (IPTG) at 20 °C overnight. Cells were harvested and resuspended in lysis buffer (20 mM Tris-HCl, pH 7.5, 500 mM NaCl, and complete EDTA-free protease inhibitor cocktail (Roche Diagnostics)), then lysed by cell disrupter and clarified by centrifugation. The 6xHis-tagged protein was isolated by Ni^2+^-affinity chromatography (GE Healthcare), cleaved with TEV protease to remove the tag, and further purified by ion-exchange chromatography (Source S, GE Healthcare). The purified protein included Grb14 residues 106-356 and seven heterologous residues (GHMASGS) on the N-terminus remaining from the TEV cleavage site. Site-directed mutagenesis of Grb14^RA-PH^ was performed using the QuikChange site-directed mutagenesis system (Stratagene), and the constructs were verified by DNA sequencing.

The expression vector pProEX HTb, harboring human H-Ras residues 1-166 and an N-terminal 6xHis-tag, was mutated via QuikChange to harbor the activating mutation G12V. The recombinant plasmid was transformed into *E. coli* BL21 (DE3), and cultures were grown in LB media supplemented with ampicillin (100 µg/ml) at 37 °C and induced with 0.5 mM IPTG at 30 °C for 5-8 h. Cells were harvested and resuspended in lysis buffer (20 mM Tris-HCl, pH 7.5, 300 mM NaCl, 2.5 mM MgCl_2_, and complete EDTA-free protease inhibitor cocktail (Roche Diagnostics)), then lysed as above. The 6xHis-tagged protein was isolated by Ni^2+^-affinity chromatography, cleaved with TEV protease to remove the tag, and further purified by size-exclusion chromatography (Superdex 75 (GE Healthcare)). The purified protein includes on the N-terminus five heterologous residues, GAMGS, remaining from the TEV cleavage site.

For SPR experiments, human H-Ras G12V (residues 1-171) was subcloned into the GST-fusion expression vector pET41 (Novagen). *E. coli* Rosetta 2 (DE3) was transformed with the recombinant plasmid and cultures were grown in LB media supplemented with kanamycin (50 μg/ml) at 37 °C and induced with 0.5 mM IPTG at 30 °C for 5-8 h. Cells were harvested and resuspended in lysis buffer (same as for His-tagged H-Ras), then lysed as above. The GST-tagged protein was isolated by Hi-trap GST affinity chromatography (GE Healthcare) and further purified by size-exclusion chromatography (Superdex 75).

For GTP loading of H-Ras G12V, 0.5 mM of the protein was incubated for 1 h on ice with 4 mM GTP in buffer containing 20 mM Tris-HCl, pH 7.5, 50 mM NaCl, and 4 mM EDTA for 1 h on ice. The mixture was then supplemented with 10 mM MgCl_2_ and incubated on ice for an additional 2 h.

### Crystallization and structure solution

Grb14^RA-PH^ and GTP-loaded H-Ras G12V were concentrated to 10 mg/ml each and mixed in a 1: 1 molar ratio. Initial crystallization trials were performed with a Mosquito (TTPLabTech) crystallization robot using the hanging-drop method (100 nl protein plus 100 nl crystallization solution). An initial hit at 17 °C with the PEG/Ion HT screen (Hampton Research)—20% (w/v) polyethylene glycol (PEG) 3350 condition, 200 mM MgCl_2_, pH 5.9—was optimized to 14% (w/v) PEG 3350, 100 mM MES, pH 5.9, 200 mM MgCl_2_, 2% glycerol, and 3% glucose. Crystal size and quality were improved through a combination of micro- and macro-seeding and sitting-drop crystallization. Crystal parameters are given in Table 1. Before flash freezing in liquid nitrogen, crystals were equilibrated in a series of stabilizing solutions containing reservoir buffer plus 10%, 15%, and then 20% (v/v) glycerol. Diffraction data were collected on beam line X25 at the National Synchrotron Light Source, Brookhaven National Laboratory and processed using HKL3000 [32]. The structure of Grb14^RA-PH^-Ras was solved by molecular replacement using PHASER [33], with the structure of Grb10^RA-PH^ (PDB code 3HK0) [12] and a structure of H-Ras (complexed to RalGDS) (PDB 1LFD) [18] used as search models. There are four Grb14^RA-PH^-Ras complexes in the asymmetric unit (solvent content 48.5%). Coot [34] was used for model building and REFMAC [35] was used for refinement at 2.4-Å resolution. The final atomic model contains, for each of the four copies of the complex, residues 106-356 of Grb14^RA-PH^, excluding residues 212-218 (RA-PH linker), and residues 1-166 of H-Ras, as well as five heterologous residues (GAMGS) at the N-terminus. PyMOL (The PyMOL Molecular Graphics System, Version 1.5.0.4 Schrödinger, LLC) was used for structural superpositions and PISA [36] was used to calculate buried surface areas.

### Surface plasmon resonance biosensor measurements

SPR binding measurements were performed on a BIAcore 2000 (BIAcore AB, Uppsala, Sweden) at 25 °C. Goat-anti-GST antibody from the GST capture kit (BIAcore) was covalently bound to the CM5 biosensor chip according to the manufacturer’s instructions. The running buffer used for the binding analysis was 10 mM HEPES, pH 7.4, 150 mM NaCl, 0.005% v/v Surfactant P20, and 2 mM MgCl_2_. GTP-loaded GST-tagged H-Ras G12V and recombinant GST alone were bound to separate flow cells of anti-GST antibody surfaces to a density of ~1000-1500 response units (RU) at a flow rate of 5 μl/min. 150 μl of Grb14 RA-PH, at concentrations ranging from 0 to 25 μM, were injected simultaneously at a flow rate of 50 μl/min into the two flow cells. Each injection was followed by a dissociation period of 180 s, in which buffer was passed through the cells at 50 μl/min, followed by surface regeneration. Background binding to the GST-coated surface was subtracted, and the average response from 150–180 s at each concentration was plotted. The resulting curve was fit using using the BIAevaluation software (v 4.1, BIAcore) to a steady-state model to extract the dissociation constant (*K*_*d*_). The *K*_*d*_ values from three independent experiments were averaged and a standard error was calculated.
